# Lectures on the More Important Diseases of the Thoracic and Abdominal Viscera; and on Eruptive Fevers, Hæmorrhages, and Dropsies; and on Gout and Rheumatism

**Published:** 1845-01

**Authors:** 


### REVIEWS.

---

Art. IV.—*Lectures on the more important Diseases of the Thoracic and Abdominal Viscera; and on Eruptive Fevers, Hæmorrhages, and Dropsies, and on Gout and Rheumatism.* Delivered in the University of Pennsylvania. By N. Chapman, M.D., Professor of the Theory and Practice of Medicine, &c. &c. Philadelphia: Lea & Blanchard. 1844. 8vo. pp. 383 and 448.

The first of these volumes was published some months ago; the other has just been issued from the press. No intimation is given that they are to be followed by others, but it may be presumed that they are the beginning of a series, intended to embrace the course of instruction delivered for so many years, by the veteran author, in the most ancient medical school in our country. Those who heard these lectures will hail their appearance in their present form for the sake of the pleasant reminiscences which they will revive, and of the stores of experience which they have again rendered accessible; and others, who only know the author by his extended fame, will be curious to find on the pages of these volumes evidences of that learning, judgment, and professional skill which have kept him so long at the head of the first of American schools of medicine. We are not about passing a critical sentence on them. Many considerations operate with us to pursue a different course, and although it is not the most summary, we doubt not, it will be the most satisfactory to our readers. We shall make copious extracts from these volumes, selecting such passages as are particularly recommended by their bearing upon practical medicine. From these a pretty fair estimate may be formed of the style, manner, and doctrines of the lectures.

The lectures embraced in the first volume treat of phthisis pulmonalis, cynanche laryngea, chronic laryngitis, asthma, angina pectoris, gastritis, organic lesions of the stomach, dyspepsia, enteritis, duodenal dyspepsia, chronic fluxes of the bowels, constipation, hepatitis, jaundice, splenitis, and congestion of the spleen. From the first lecture, which covers nearly a hundred pages, we shall make no extracts. Pulmonary consumption, the experienced lecturer remarks, is a disease on the consideration of which he "enters with little satisfaction;" and most writers would join him in the remark. He doubts whether a cure of the disease, when fully established, was ever effected. "Not an instance at least," he adds, "have I seen, and believe, that those who report to the contrary deceive themselves, or the truth is not in them."

An account of cynanche laryngea follows the long discourse on phthisis pulmonalis. Having alluded to the fact that it was of this affection that Washington died, and that it is a disease of advanced rather than of early life, he thus speaks of its pathology:

"Embarrassment as to the pathology of laryngitis is still confessed by many, though really I cannot perceive any peculiar obscurity about it. Enough, at all events, is ascertained, to assure us of its seat being mainly in the upper portion of the windpipe; and that, the action usually partakes of a mixture of spasm, and inflammation is no less apparent. But, in this respect, there is a striking similitude to croup, in the early stage, the most remedial of diseases:—whereas, laryngitis proves directly the reverse! To solve this problem satisfactorily, has perplexed some of the ablest writers. Towards an explanation of it, I will suggest, that much of the peculiarity of the latter affection as well in relation to the symptoms, as the difficulty of cure, proceeds from the position and nature of the phlogosis. That of croup, restricted more especially to the mucous tissue, is adhesive, eventuating usually in the exudation of coagulable lymph on its surface. Expending its force on the contrary in laryngitis, chiefly on the subcellular membrane, it is of another character, productive of tumefaction, ending sometimes very speedily, and often when protracted, in effusion of serum, or lymph, or the secretion of pus, the first being the most common event. Laryngitis, in a word, is an affection, having an identity with the inflammation of common sore throat, in every leading feature, and is more serious only, from its location in a vital structure. It is very intelligible how either of its ultimate states mentioned, influences the case. By swelling from phlogosis alone, the larynx may be obstructed—and to this condition, the peculiar phenomena of the affection are mostly referable. But it is occasionally otherwise. The cellular which lies under the mucous membrane, becoming distended by an effusion, pressure is made on the rima glottidis, approximating its sides, thus occasioning a similar mechanical interception to the passage of air to the lungs."

After speaking of the use of venesection and emetics as remedies adapted to the first stage of this complaint, he mentions tobacco in the following terms:

"Nothing, however, within my experience, and I have tried divers means, so completely controls these periodical exasperations as a cataplasm of tobacco around the neck, or smoking the article, where the individual is not habituated to its use. The success indeed, of the few experiments I have made with it, leads me to believe, that it may prove a very important therapeutic agent in the more general management of the disease. It occasions very severe and protracted nausea, followed by thorough relaxation, and probably abatement of phlogistic action. Nausea, when long endured, has, indeed, a very decisive control over all such conditions, and particularly when there is a combination of spasm with inflammation. Examples are very numerous and strking to this effect. Colic is one of those instances, and I have seen paronychia, even furunculus, seated in the cellular texture, itself, mitigated or cured by it."

The eminent author is no friend to lobelia. He says:

"Many speak favorably of the lobelia inflata, and some extravagantly—with which I have no experience. From its employment I have forborne, on account of its deleterious properties as exhibited in the administration of other practitioners, which I long ago witnessed. Of these poisonous articles I am terribly afraid, and should really experience some 'compunctious visitings of conscience,' were I to perpetrate death by such hazardous experiments."

The facts adverted to in the following extract are curious, and calculated to excite much interest. Treating of gastritis, the author remarks:

"There are, however, several points which are still left in some obscurity.

"1st. What is the explanation of the fact now admitted, that the most violent gastritis may prevail without its being betrayed by any symptoms whatever? It is probable, that, in most of such instances the stomach, having sustained a loss of its organic sensibility, it feels not its injuries, or, at least, does not manifest them—though on other occasions, the gastric lesion may be masked by the preponderancy of the affection of another organ, and above all the brain. Be this as it may, there is no peculiarity in the occurrence, every other viscus of the body, the lungs, the liver, the spleen, the kidney, the brain, &c., sometimes disguising the severest of even its structural derangements, in the same way.

"2d. To account for the phenomenon, that not the slightest inflammation can be detected in the stomach, when every assurance by symptoms had been given of the existence of gastritis, has proved equally perplexing. Different explanations does it admit. It may be ascribed to the reaction of the blood from the capillaries, by the act of death, as happens in the skin when inflamed by the exanthemata—and also, in the serous tissues, the peritoneum or pleura, of which some striking examples are recorded—or it may be referred to the emptying of the vessels by the copiousness of the effusions. Confirmed in some degree, is the latter hypothesis by the consideration, that in the pestilence to which I have alluded, whenever an excessive excretion of black vomit took place, no phlogosis or congestion was observable. To more common cases of gastritis, there are equally incident sanguineous, serous, and other exhalations, which may be productive of a similar effect. Nor is it improbable, that it sometimes is owing to such an overwhelming impression from the operation of the cause, as to prevent inflammatory action, though otherwise, the organ may fatally suffer, as in the case of prussic acid, &c., or to the disease of the stomach having entirely ceased by a translation of it to some other part.

"3d. As to the circumstance of the stomach exhibiting the appearances of phlogosis, when it could not possibly prevail, the explication is more easy.

"In most cases of sudden death, and particularly in those I have referred to, it appears sufficiently, that it is occasioned by asphyxia, which state we know, independently of the direct evidence of Bichat, from actual inspection, produces fullness of the capillaries. Why this happens oftener and more prominently in the stomach, as is represented, probably proceeds from its being the *ultimum moriens.* Hallar states that vitality is sensibly retained in it, and the small intestines, when extinct in every other part. Conclusively, however, the late experimentalists have observed, that in opening living animals, for the purpose of physiological investigations, they uniformly perceived the stomach to become a centre of fluxion—its vessles filled, and its surface reddened, or darkened, exactly in proportion to their struggles. No structure, indeed, is so prone to assume these appearances as the alimentary canal. During chymification, the stomach has been proved to be red, or turgescent, from an augmented flow of blood to it—the same condition is presented by the duodenum, when it is engaged in the digestive process, and by those portions of the large bowels, where the fæcal matters accumulate and for a time are detained. The distinction between gastritis and gastric fever, consists mainly in the one being local, and the other so wide a sympathetic extension of the irritation, as to embrace and disorder more or less of the system, and especially the capillaries, which last is the essence of genuine fever It is a fact too interesting not to be pressed on attention, that the tendency to diffusion, in this case, is in the inverse ratio of the intensity of the primary phlogosis."

The learned professor thinks that physicians sometimes give their patients dyspepsia by excessive drugging. His statement of the case, which is as follows, is a strong one:

"Nor can the profession escape the imputation of lending its contribution to this mischief. Called to a case of disease of such obscurity, that no distinct notion can be formed of it, we go on groping in the dark, pouring down drugs empirically or at least tentatively, till the stomach gives way, and its derangements are added to the pre-existing affection, by which a case is made of greater complexity, and of enhanced difficulty to cure. It is not easy always to avoid this course, from the ignorance or prejudices of mankind. The predominant estimate of the profession, even among the most enlightened people, leads to the delusive supposition that the materia medica has a remedy for every disease, and that the want of success under any given circumstances, is owing to the poverty of resource of the practitioner in attendance. Confidence is soon withdrawn should he intermit his exertions, which perceiving, he too often multiplies his administrations to avoid a dismissal, or to have imposed on him some one of the fraternity who, it is expected will bring forth fresh supplies. The consultation taking place, the new armory of weapons is opened and applied, with only an exasperation of the case. Not satisfied, however, further trials of others are made—there is a repetition of a similar proceeding, and the catastrophe is completed. This, which might by some be suspected as a sketch of fancy, is a faithful and unexaggerated delineation of a reality, I have frequently seen and delpored. Convinced that he was falling a victim to this very practice, the Emperor Hadrian deliberately prepared as an inscription for his tomb:

"'It was the multitude of physicians that killed the Emperor.'"

Of tobacco, as a cause of dyspepsia, the author has the following to say:

"The most common of the causes of the disease, in certain parts of our country, is the enormous consumption of tobacco in the several forms. Certain I am, at least, that a large proportion of the cases of it, which come to me, are thus produced. It is usually very obstinate, and sometimes of a truly melancholy character. Easy as it were to cite numerous instances to this purport, I must be content with a limitation.

"By a member of congress from the West, in the meridian of life, and of a very stout frame, I was sometime since consulted, who told me, that he labored under the greatest physical and moral infirmity, which he was utterly unable to explain, and that, from having been one of the most healthy and fearless of men, he had become, to use his own phrase, 'sick all over and as timid as a girl.' He could not present even a petition to congress, much less say a word concerning it, though he had long been a practising lawyer, and served much in legislative bodies.

"By any ordinary noise he was startled, or thrown into tremulousness, and was afraid to be alone at night. His appetite and digestion were gone—he had painful sensations at the pit of the stomach, and unrelenting constipated bowels.

"During the narrative of his sufferings his aspect was ghastly, approaching the haggard wildness of mental distemperature. On inquiry, I found, that his consumption of tobacco was almost incredible, by chewing, snuffing, and smoking. Being satisfied that all his misery arose from this poisonous weed, its use was discontinued, and in a few weeks he entirely recovered.

"Distressing as was this case, I have seen others, from the same cause, even more deplorable. Two young men from the country were in succession brought to me for advice, whom I found in a state of insanity very much resembling delirium tremens. Each, I was told, had chewed and smoked tobacco to excess, though perfectly temperate as regarded drink, and which representation proved to be correct. The farther account given me was, that early in life, adopting this bad practice, it grew with their growth. Dyspepsia soon occurred, attended by great derangement of the nervous system, and ultimately the mania I have mentioned. But I have also seen the same condition very speedily induced.

"Five years ago, I was requested to visit a young man at one of the hotels in this city, who had been a resident in it for several weeks. The general aspect of the case so strikingly resembled delirium tremens, that I at once pronounced it to be that affection. But I was assured of his sobriety, and that he had been brought to his present situation by having kept his bed for several days, under the influence of nostalgia, during which period he actually subsisted on tobacco. More recently, I saw quite a youth affected in precisely the same manner, owing, most unquestionably, to the exorbitant use of cigars. Both of these cases were cured chiefly by abstinance from this pernicious practice."

Among the causes of chronic hepatitis Professor Chapman enumerates the excessive use of mercury. His words are—

"Nor can I help thinking, and on no slender grounds, that the extravagant use of mercury by many of our practioners, in the treatment of autumnal fevers and other diseases, must also be assigned as frequent cause of chronic hepatic affections in some portions of the United States. More than any other article of the Materia Medica, at least, has it the power of exciting the actions of the liver, and it is a law of our nature, that all high excitement is followed by a correspondent degree of debility. From the circumstance of the prodigious employment of calomel in cases to which I have alluded, amounting to several drachms daily, it seems to be no unreasonable supposition, that the hepatic apparatus, thus over-stimulated, should fall into collapse, and, in this condition of exhaustion, torpor to take place in the portal circulation, productive of congestion, eventuating in phlogosis, induration and other derangements. Doubtless in this mode do miasmata and high temperature, separately or unitedly, and the habitual consumption of ardent spirits operate to the same effect. As confirmatory of this view, it is stated, by Dr. Somervail, a most respectable physician, of the South of Virginia, who has practised medicine for nearly half a century, in that section of the country, that till the introduction of mercury, a comparatively modern event there, into the treat- of autumnal diseases, hepatitis was hardly known, and subsequently, it has most widely prevailed. We have also seen, that it is the opinion of Annesley, whose opportunities of witnessing the effect were so ample, that the large use of mercury in the inflammatory stage of acute hepatitis, by an increase of excitement, has a tendency to induce chronic degenerations. That it occasions jaundice, most probably, by deranging the liver, will hereafter appear, as well on the authority of some cases which several years ago occurred to myself, as others, more recently reported, by highly respectable European writers, in support of the conclusion."

A large space in the second of these volumes is given to the consideration of *small pox*, and its prophylactic, vaccination. The author accumulates a large amount of testimony concerning the efficacy of the vaccine virus, and he thus sums up in conclusion:

"It seems, on the whole, to be agreed among those who have had opportunities of judging, that the primitive matter, wherever obtained, proved more active and operated with greater certainty. The local inflammation, as well as the constitutional disorder, was more violent, and a manifest improvement exhibited in the vesicle and subsequent pustule, areola, &c. How far this representation is correct I cannot say from my own observations, every attempt which I made with the Bristol matter having been unsuccessful.

"The experiments to which I have alluded are very defective. They show only greater energy in the new virus, leaving the fact of the capability of the impression created by it to protect against small-pox immediately or remotely in absolute doubt. Nor does the mere circumstance of higher intensity of action, local or general, or even of more perfect development of the pustule prove very conclusive. The spurious disease, in many instances, as previously intimated, is remarkably distinguished by the two former particulars, and the occasional fallacy of an unexceptionable local affection, in every respect, as to its commencement, progress, maturity, and declination, is universally admitted. Examples, without number, of the failure of vaccination, and when performed, too, by the most experienced, have occurred with every favorable indication. Much remains to be accomplished in this investigation to the ascertainment of truth."

Measles is the subject of the next lecture and we again extract the closing paragraphs, which relate to points of practical interest.

"Measles is singularly prone to sequelæ or consequent affections, among which are catarrh and pneumonia, opthalmia, aphonia, diarrhœa and some other more chronic derangements. Even when a predisposition only exists, it is exceedingly apt to ripen the tendency into actual disease, and particularly scrofula and phthisis, to which some writers add hepatitis. Nothing, however, very peculiar characterizing the management of the generality of these secondary affections, I shall scarcely notice them further. It may be sufficient to say that the whole of them are to be treated by those remedies the best adapted to subacute or chronic inflammation, which is their true pathological condition. Yet diarrhœa, from the extreme debility often associated with it, is apt to lead to a deception, and which, on this account, may require to be specially designated.

"Too commonly, under such a delusion, a resort is had to the astringent and testaceous preparations, which are both ineffectual and mischievous. The lax, in this case, is the result of inflammation of the mucous coat of the intestines, and can only be relieved by the measures I have intimated. It was Sydenham who first made known this fact, and subsequent experience has abundantly confirmed it. Bleeding, general or topical, in moderation, with ultimately a blister to the abdomen, and the Dover's powder exhibited at night, with an occasional recurrence to the warm bath, constitute the proper means. Continuing obstinately, however, calomel or the blue pill, in minute doses, with opium or otherwise, becomes necessary, and more especially where the liver is concerned, which sometimes happens.

"It remains to remark, that the black appearance occasionally taking place at the close of the eruption, giving to the disease the title of rubeola nigra, is said to be speedily removed by the internal use of the muriatic acid. But, for the most part, as spontaneously subsiding in a short time, it may be disregarded.

"My wish, in closing the subject, is further to urge the importance of more circumspection in the treatment of measles than it usually receives. We have seen that, in its graver states, how many organs it involves and the serious injuries inflicted. As much, perhaps more to be dreaded, are such lesions than the disease itself. That the affections it entails or developes are the results of imperfect cures, and hence might be obviated by better practice, is my deliberate opinion. Being decidedly inflammatory in its common nature, it obviously exacts the antiphlogistic course in all its details, and especially the loss of blood, to the neglect of which I am disposed to ascribe mainly the mischief so lamentably experienced. Never have I known a case of inflammatory measles to resist adequate bleeding. But our care must not stop here. Caution should be enjoined, even after the disease is seemingly cured, and still more during convalescence, against any exposures to cold, or improper indulgencies, or trespasses of any kind."

Scarlatina is a malady, the mention of which will excite a much deeper interest—it is treated of in the succeeding lecture. Concerning its history the author thus expresses himself:

"Doubtless scarlatina is a disease of modern date. Certain passages have been cited from some of the ancient writings, thought to refer to this, and its affiliated affections, which, on a more careful examination, will not bear such an interpretation. Its introduction into Europe is commonly traced to an importation from Africa, and is said to have first broken out in Spain, in 1610, whence it spread to Naples, where it prevailed eight years afterwards, as an epidemic, with unexampled violence. Fifty thousand persons are said to have died of it in that city, and half a million in the Neapolitan and adjacent territories, before its cessation.

"By Sydenham, and subsequently Moreton, some account was given of it as prevailing in London in 1689. But so widely do their descriptions differ, that they can hardly be supposed to refer to the same disease, the one representing it to be very mild, and the other as directly the reverse.

"In 1735, it made its appearance in New England, gradually diffusing itself, to a greater or less extent, over this continent. Much confusion, however, exists in the history of the disease, owing to the want of a just discrimination in those by whom it is described, and especially at an early period. No distinction is made in most of these records between it and measles, or even small-pox, except as to gradation of violence; and so lately as the time of Moreton, and even of Watson, the latter of whom wrote about the middle of the last century, the identity of it and measles was maintained. Bateman declares 'that Withering's publication of it in 1778, or rather the second edition of it in 1793, may be considered as the date of the correct diagnosis of the disease.' "

The author believes in the contagiousness of the disease. He says—

"It is questionable at what stage scarlatina acquires this property, prior to or after the eruption. Many believe that it is most active during the desquamation, and at all events, it seems to retain it till this process is completed—the scales being impregnated with the virulent secretion of the skin. Yet the disease cannot be propagated by inoculation with matter procured from this or any other source. Contrary statements have been made, I am aware, though on no good authority. To *fomites* of various kinds it adheres very tenaciously for a long period, with an entire preservation of its efficiency, in proof which we have some striking facts. My friend, the late Dr. Percival, of Dublin, imputes the introduction of scarlatina into that city to a box of toys from London, which had been exposed to the contagion. That the disease was propagated in this way, however, is hardly to be credited, and the story seems to me very like the famous report of Hildebrand, who assures us, that as soon as he arrived in Padolia, scarlatina broke out and spread most widely, which he ascribes to the retention of contagion in a coat he had worn in the disease a year previously in Vienna. Nevertheless, the long continuance of infection in apartments where the disease has existed, though every purification be practised, is unquestionable. Elliotson, of London, states, in confirmation of it, "that all the children admitted into a particular ward in a hospital under his care, were seized with scarlatina for nearly two years, in consequence of a patient with the disease having been in the ward at that remote period, and this in despite of white-washing and other cleansings.' "

We give his remarks on belladonna as a preventive of the disease, the last of which are highly characteristic of the author:

"As a preventive of scarlatina, the use of belladonna has, of late, attracted some attention. The facts, indeed, bearing on this point, are exceedingly curious, and, coming from respectable sources, ought not to be contemptuously or heedlessly passed over or rejected. Berendt, a physician of Vienna, declares, that, by the use of the article, only fourteen out of one hundred and ninety-five children exposed to the contagion, took the disease, and these had it very mildly. We are further told by Professor Herholdt, of Copenhagen, that he found it to preserve several hundred children, during the prevalence of the disease as an epidemic in that city; and, on a subsequent occasion, when it appeared even more violent, out of nearly an hundred families, all escaped except one, and of this he is doubtful whether they took the medicine. The testimony of Koreft, of Berlin, is scarcely less decisive—that of Godelle, who avers that *he has never seen it to fail*, still more so, and a variety of attestations from other medical men of the continent of Europe, might be adduced. Ten or fifteen drops to children, according to the age, morning and night, of the watery solution of the extract of belladonna, in the proportion of two or three grains to an ounce of water, is the common mode of exhibition.

"To what extent these statements are to be credited, I cannot say from any experience of my own. Distinct from the authority by which they are sustained, when we advert to the powerful impression of the belladonna, we may, on the principle of the incompatibility of two actions simultaneously existing, get an explanation of its *modus operandi*, and be not altogether incredulous. That preoccupying the stomach by food, cordial drinks, and even by certain medicines, as opium, or bark particularly, has proved prophylactic as to intermittent and some other diseases, is abundantly demonstrated. Yet, by the assurance of Hahneman, the author of the homœopathic doctrines, which, originating in fraud and imposture, and continued by the most atrocious wickedness, that he was equally successful in the infinitesimal dose of a few drops, daily, of a solution, each drop of which contained no more than the twenty millionth part of a grain of the extract, distrust and even ridicule are cast over the whole affair, and our faith must be withdrawn or suspended till fresh and better evidence is afforded."

Following out our plan of presenting extracts from each lecture, we have next to give one from that which discusses hæmorrhage at great length. As usual, the part selected contains practical suggestions—it relates to the use of emetics in hæmoptysis:

"It remains only to detail some of the results of my experience, in confirmation of the efficacy of the practice which has been suggested, to be illustrated by a few examples.

"In 1807, I was called to a young man of consumptive tendencies, who, for several months, had suffered occasionally from hæmoptysis, and was treated by another physician in part by digitalis. Being suddenly attacked with a copious effusion of blood, he took before my visit an exorbitant dose of the medicine which excited vomiting; the hæmorrhage ceased, he became convalescent, and ultimately recovered. Effects so decided I did not then impute altogether to the act of puking. As often happens from digitalis, an extremely distressing nausea continued for several days, to which I thought it probable the permanent benefit was in a considerable degree owing.

"Encouraged, however, by the cure, and influenced, perhaps, still more by theoretical notions of the nature of hæmorrhage, and of the applicability of the remedy to it, I resolved to subject the practice to a further and fairer trial.

"It was not long before I had an opportunity of doing so, in the instance of a young man from the country, whom I had been attending for several weeks, for pulmonary consumption. Three years previously to my seeing him, he was compelled to abandon the study of the law on account of the frequent recurrence of spitting of blood—and when he came under my care was far advanced in phthisis. One night he was aroused from sleep by a repetition of the hæmorrhage, and on my arrival had lost more than a pint of blood without any diminution of the flow. Common salt, sugar of lead, and such like articles were used in vain; and bleeding, generally or locally, seemed inadmissible, from the debilitated state of the system, and of the pulse especially. Excepting an emetic I was nearly destitute of resource in this emergency; and accordingly twenty grains of ipecacuanha were administered, which soon bringing on vomiting, the effusion was suppressed. On several subsequent occasions the same means proved equally effectual, though ultimately he died of the main disease.

"I had within a month a third case, which afforded me a further opportunity of pursuing the practice, and of confirming my confidence in its efficacy. It was that of a young woman, who, a considerable time before, having suppressed her menses by an exposure to cold, had, ever since, at irregular periods, suffered from hæmoptysis. When I saw her, which was in consultation, the hæmorrhage had already existed for forty-eight hours, the loss of blood very considerable, and she much exhausted. As the usual remedies had been unavailing, I induced Dr. Stewart, with whom I was attending, to try an emetic of ipecacuanha, which put an end to the hæmorrhage, and by propor management, subsequently menstruation returned, and with it a restoration of health.

"More than thirty years have since elapsed, during which lengthened period I have pursued this treatment, and with such success as to have inspired great confidence in it. Like all other means, it will sometimes fail, as might be expected from the diversified nature of the causes and states of the hæmorrhage. But I am persuaded, when properly applied, it will be found to do more than any thing else, and certainly from my own observations, it never produced mischief in the vital or spontaneous extravasations. The emetic I have preferred, and, indeed, only prescribed in these cases is ipecacuanha."

We shall offer longer extracts from the discourse on *purpura hæmorrhagica*—an affection, the causes and pathology of which are still involved in great obscurity. On the former point our author remarks:

"Not much is satisfactorily determined as to the causes of purpura. The highest authorities, however, seem to be agreed that it is mainly associated with a bad habit of body, acquired by some positive antecedent disease, or by inhabiting low, damp, dark, ill-ventilated dwellings, with the other incidents inseparable from poverty; penurious diet, defective clothing, labor to fatigue and exhaustion—all aggravated by mental anxiety or sharper misery. But no less is it admitted, that the affection occurs, and, perhaps, nearly as often among persons seemingly in sound health, and in the full enjoyment of the comforts and felicities of life. These are the representations of the books, which I am inclined to suspect refer to two states of disease not identical—the one a modification of scurvy, and the other purpura proper—thus indiscriminately confounded. Much have I seen of the former, in the final stages of cholera infantum especially, and only some five or six cases of the latter—every one of which was in a healthy subject, of respectable condition, with no privations or inconveniences of living.

"The principal difficulty in the diagnostication of purpura arises, indeed, from its analogy to these scorbutic bleedings. Contrasting, however, the two affections in their prominent features, we shall perceive sufficient individuality in them without descending into details. Genuine purpura seems to me to be of sudden access—often without an appreciable cause—takes place in sound individuals, and is a singularly pervading and an active hæmorrhage—the dermoid tegument participating to a limited extent only. Extravasations of blood incident to scurvy, are reversely slow in development, uniformly preceded by extreme pravity of system, brought on by obvious causes, are more local and of smaller amount, the blood being also different. Yet, much perplexity existing, the whole history of the case must be surveyed in its rise, progress, and present condition, so as to render a correct decision attainable."

He has not the difficulties which many experience as to the pathology of the disease. His views concerning it are conveyed in the following extract:

"The peculiar obscurity confessed by many in the pathology of purpura, I cannot perceive. That it is not of the nature of scurvy, nor of the petechiæ of fever, is clear. Neither can it be placed among the exanthematous eruptions. As an hæmorrhage it must be considered, varying from others in this respect chiefly, that while the generality of them are assignable to obvious causes, this one comes on in the absence of every thing to explain its production. Coupling this circumstance with the extensiveness of its prevalence over the body, I know not to what else it is referable than the constitutional state, vaguely called the hæmorrhagic diathesis or tendency. Great reason have we to suppose that it is independent of any of the ordinary organic lesions, and proceeds from derangement of innervation, producing the change in the capillaries and in the blood itself, favoring sanguineous effusions, and which condition may be natural or acquired, the latter, sometimes, also permanent or very enduring."

We copy his remarks on the treatment of purpura:

"Called to a case of any activity, venesection should be instantly performed. It is the only decisive remedy. Nor is the amount of blood detracted to be small, or any timidity to deter from a repetition of the operation, where the necessity for it exists. I drew as much as sixty ounces in one case, and in another half this amount, in several successive bleedings, with the most decisive advantage. The course is justified by the state of the circulation, by the appearance of the blood, which is thick and sometimes heavily sized, by the albuminous urine, by the acuteness of pain in the cavities, and by the relief afforded from the hæmorrhage itself when not excessive. Many of the European practitioners, as Parry, McIntosh, &c., give to it their support, though not so intrepidly as I have ventured. But it is not to be supposed that under any other circumstances than stated, and in the extremest emergencies, would I carry it to such an extent. Generally, my bleedings are far more moderate, and especially in an advanced stage, where the loss of blood is ill borne.

"Next in value of the remedies is purging deemed, with the saline articles, and there are facts to render it probable that the sulphate of soda is specially adapted to the disease. Great benefit, however, is derived by cold applications to the surface—either sponging or covering it over with cloths wrung out of water of a moderate temperature. Nay, on one occasion, in consultation with Dr. Meigs, we immersed a girl in a cold bath, and with such effect that henceforward she speedily recovered. It was on the second day of the attack—the purpura pervaded her skin, and though she had been previously depleted, the pulse was still full and strong, and the surface warm.

"No confidence do I place in the astringents and acids usually appropriated to hæmorrhage, and have little experience of the alkalies and neutral salts recently recommended on the authority of Stephens. But I am disposed to believe that opiates are deserving of attention, more, however, from analogy than any better evidence. Emetics and the spirits of turpentine I would, for the present, place on the same footing.

"In the less active states of this disease, some variation in the treatment is required. Blood-letting, if admissible at all, must be practised cautiously, as well, indeed, as whatever else is calculated to increase debility. Debarred of the former, however, we are nearly destitute of means of any certainty of advantage in an urgent emergency. Those previously enumerated, and commonly employed, have proved nugatory in my hands. My trust would be mainly in the sulphate of soda, the spirits of turpentine, the sulphate of quinine, opiates and wine."

The following extract is hygienic in its character, and will be read with pleasure. The testimony borne in it to temperance by the veteran professor is emphatic and consolatory. He is treating of gout, the connexion of which with particular modes of living is well understood. He says:

"Of the dependence of gout on the habits of living, no stronger proof can probably be supplied than from the annals of this city. When I commenced my professional career, the disease abounded in the higher circles, and then it was the practice to drink punch in the forenoon, to continue it at dinner, or to resort to ardent or malt liquors, followed by a liberal use of diverse wines, closing the evening with substantial suppers and stimulating potations. But, in this respect, within the last thirty years, a signal change has taken place. No punch or distilled spirits, and comparatively little malt liquor, has been consumed, and the custom of supping is nearly extinct. Temperance has superseded debauchery or excess, and gout, thus deprived of its aliment, is fast perishing away. My opportunities have enabled me to ascertain the fact, that so late as the commencement of the present century, a hundred cases of the disease existed in this community where one is now to be met with, and, with few exceptions, these are the remnants of other days, serving as memorials of a state of society, of which there are scarcely any other traces to be recognized. Literally is it true, as expressed by May, one of the oldest of the English dramatists:

> "From our tables now, no painful surfeits,
> No fed diseases grow, to strangle nature,
> And suffocate the active brain. No fevers,
> No apoplexies, no palsies, no gouts
> Are here."

"Contemplating this happy reformation, who can forbear to exclaim with the fair poetess—

> "'Tis to thy rules, O temperance! that we owe
> All pleasures which from health and strength can flow;
> Vigor of body, purity of mind,
> Unclouded reason, sentiments refin'd,
> Unmixt, untainted joys, without remorse,
> Th' intemperate sinner's never-failing curse."

"To the causes of gout must, as previously intimated, be added sedentary, indolent, or intensely studious habits.

"It is well known, that in the common orders of life, the disease hardly exists, and infinitely less among the higher classes who pursue occupations of active industry. Neglect of the exercise of walking has particularly an effect, and among other evidence of it, which we learn, is, that while the people of Edinburgh, who, from the location of their trade at Leith, the seaport town, a mile or more distant, usually ride, are very liable to the disease; those of Glasgow, the practice of whom is different, are nearly exempt from it.*

"Not doubting the influence of this custom, to a certain extent, I am still inclined to impute more to a wider difference in the character and habits of the inhabitants, in other respects, of these two cities. Edinburgh is the abode of the opulent, the noble, the learned, and also the refuge of nearly every veteran of Scotland, of the military, naval, or civil service, who returns from the performance of arduous and lengthened duties, in distant and sickly climes, with shattered constitutions, to enjoy at home luxurious ease. The one city

* Scudamore on Gout.

has claims to the highest intellectual society, though voluptuous, and even grossly dissipated, at least it was so in my time, and the other the reverse, or that marked conspicuously by economical prudence and active, stirring, money-making industry. Thus contrasted, can the disparity, as to the relative prevalence of the disease in the two places, be a matter of surprise?"

The following extracts exhibit the author as historian. In the first he is relating the process of *inoculation*, and in the second the origin of *vaccination*. Such passages enliven many of his pages, and amid the dry details of practice must have proved eminently refreshing to his pupils. These extracts afford fair specimens of the style of the author which is not without its peculiarities. Having noticed the slow advances of inoculation in England, he goes on to say:

"The progress of this discovery was, throughout the continent of Europe, still more retarded. Except in Hanover, into which it had very partially crept, no where was it to be recognized. *Narcotized* Germany laid, at the time, prostrate under the stupefaction of despotism, unable to 'shake the poppies from her head,' insensible to improvements, and, perhaps, without the consciousness of possessing those fine intellectual energies she has since displayed. The very attempts to introduce this new practice were met by mounds of popular prejudice, encouraged and fortified by her most eminent medical men, De Haen, Van Swieten, &c.

"Even in France, with the illumination of her literature, science, and philosophy, it had to encounter, for a season, an irresistible opposition. Condemned, *ex cathedra*, by the faculty of medicine at Paris, it remained slighted for many years. Neither appeals to reason nor common sense, nor the weight of cumulative facts, had any effect. Finally, however, Voltaire, returning from England, whither he had been, took the subject in hand, and, by his representations of the immense advantage of the new practice, which he had there witnessed, and above all, as the only preservative of female beauty against the most hideous deformities, enlisted the ladies of the court in its behalf, and henceforward inoculation became quite the vogue in the higher circles, whence it spread, as other fashions, among the people. To the influence of this extraordinary man, operating on the vanity of women, does it appear that we are mainly to assign the just appreciation of one of the greatest boons ever conferred on humanity—such being the mysterious modes often adopted by Providence for the revelation or dispensation of infinite goodness! Means, however, were still wanting to execute the plan which had been devised of extending this blessing to the poor, and these were supplied on the coming into power of Turgot, the wise, the liberal, the benificent minister of the French treasury. Enough, perhaps, has been said to show that this, in common with every other improvement, wheresoever it may emanate, was eagerly received, and skilfully pursued by our own, in the proper sense of the term, truly enlightened country, whose mind has never been perverted by vulgar prejudice, or weakened by fearful superstition, or its determinations thwarted by aristocratic influence, or the power of antiquated corporations, or that of Government itself, so perniciously felt in the Old World."

The history of Jenner's immortal discovery is thus pleasantly mixed up with anecdote, and we give it without any fear that it will prove tedious, although the extract is a long one:

"In 1768, while Jenner was a student of medicine, residing in Gloucestershire, he heard of the prevalence of an affection on the udder of cows, of a pustular nature, which occasionally infecting the hands of those who milked them, produced a correspondent affection, proving a preventive of small-pox, as well in the natural way as by inoculation.*

Deeply impressed with the value of the intelligence he had collected, Jenner repaired to London, to prosecute his studies under the auspices of John Hunter. During the period of their connection, frequent conversations were held between them regarding the cow-pox and its reputed properties. Engrossed, however, with other occupations, the preceptor was

*Not uninteresting can it be here to mention, that I have been told by Dr. Neil, of this city, that much about the same period, and certainly without any information from abroad, his father, who practised physic on the Eastern Shore of Maryland, was in possession of essentially similar facts. It has also been rendered probable, by M. Hussen, that a certain M. Robant, Protestant minister at Montpelier, in France, had so early as 1781, made some verbal communications to several persons, indicating considerable knowledge of the subject.

without leisure, or did not deem the matter worthy of his immediate attention. Having returned home, after a considerable interval, Jenner resumed his favorite investigation, which he steadily pursued till the consummation of his mighty and marvellous discovery. This took place on the 14th of May, 1796, which is held to be the birth-day of vaccination. It was on that day, says his biographer, Barron, 'that matter was taken from the hand of Sarah Neimes, who had been infected by her mother's cows, and inserted by two superficial incisions into the arms of James Phipps, a healthy boy of about eight years old. He went through the disease apparently in a regular and satisfactory manner. But the most agitating part of the inquiry remained to be performed. It was needful to ascertain whether he was secure from the contagion of small-pox. This point, so full of anxiety to Jenner, was fairly put to issue on the 1st of the following July, when inoculation with variolous matter, immediately taken from a pustule, was practised without any infection.' But though, having thus experimentally determined the fundamental principle of his discovery, he still sedulously proceeded to perfect it by the investigation of details, or of collateral matters, calculated to illustrate and confirm it.

"The points which he thought he had ultimately demonstrated were, that the cow-pox was readily imparted to the human species by inoculation, to be passed from individual to individual, without deterioration or change, giving an immunity against small-pox—it being itself a very mild affection, entailing no pernicious consequences, and utterly destitute of the quality of infection through the medium of the atmosphere, or of communication in any other mode than the one stated.

"Entirely satisfied of the accuracy of his conclusions, he resolved to lay them before the public through the transactions of the Royal Society of London. That learned body, however, placed such slender confidence in his representations, so extraordinary did they appear, that his paper was returned, with a friendly admonition to withhold it from the press, lest it should injure his reputation. Happily, the advice was not followed; and in 1798, twenty years after the commencement of his inquiries, he published, in a small pamphlet, in a style the most modest, his great discovery. From this period, vaccination came progressively to be diffused throughout the civilized world, meeting, in different countries, various degrees of resistance, according to the extent of prejudice against it, or the inefficiency of exertion in its behalf.

"More at home, however, than elsewhere, was it assailed by vulgar or illiberal abuse, pretty much in the mode previously pursued in regard to inoculation. Conspicuous among the anti-vaccinists was Mosely, advantageously known by his writings on tropical diseases, whose hostility amounted to fanaticism. Determined in his opposition from the first, to the practice, he ultimately issued a small tract upon the subject, in which, amidst other extravagant absurdities, he ventured the prediction that, 'by the influence of bestial humors, imparted in the process, the 'human form divine' would become degenerate, and another brood of Minotaurs produced.'

'Semibovemque virum, semivirumque bovem.'

"Mosely had occupied a considerable position in the professional and social relations of London, and his perpetration of such folly created much surprise. But, as I have heard, he was actually insane at the time. From the patronage of the Duke of York, he had lavished on him the practice of the court, and higher circles, with the lucrative sinecure of physician to the Chelsea Hospital. Thus, as he was basking in the sunshine of prosperity, an incident occurred to blight his fortunes. The Duke having a cold, sent for Mosely to consult him on the safety of his going out to review a body of troops lately returned from the Egyptian expedition, who protested against his leaving the house.

'The dawn was overcast, the morning lower'd, and
Heavily in clouds brought on the day.'

"Nothing, however, could restrain the ardor of the hero, and while on the *deluged* field, he broke out with measles, and became very ill. The physician was charged with criminal ignorance, for not having promptly discerned the nature of the inchoative disease that jeoparded the life of his royal highness, and was instantly punished by deprivation of all his privileges, honors, and appointments. Calamities so heavy, and unexpected, were insupportable. His mind gradually gave way under the pressure of grief and mortification, and, while in a state of hallucination, this unhappy production escaped from it. Not long afterwards, he died in poverty, obscurity, and confirmed madness:

——————— 'O, how wretched
Is that poor man that hangs on princes' favors!
There is, betwixt that smile we would aspire to,
That sweet aspect of princes, and their ruin,
More pangs and fears than wars or women have,
And when he falls, he falls like Lucifer,
Never to hope again.'*

"But, though subjected to this and some other annoyances proceeding from individuals of greater responsibility, actuated by ignorance, envy, or malice, Jenner was encouraged and sustained by the applauses of the better part of his profession, the countenance of his sovereign, the liberal rewards of Parliament, and the gratitude of mankind. Many of the most eminent personages of the period courted his personal acquaintance, and nearly every learned association enrolled him among its members, or bestowed on him its honors. Not to swell the catalogue of his titles, he was elected into the Royal Society of London, the National Institute of France, and the American Philosophical Society; and received the degree of Doctor of Laws from Oxford, and one or more of our own universities. Both hemispheres united in rendering him its grateful homage.

"Napoleon himself, at the height of his glory, bowed to the supremacy of his merits, in an offering of a tribute to them deserving of preservation, from its moral beauty. It happened that, among the Englishmen detained at that moment in France, in retaliation of an act of hostility of the British Government, which had engendered the most envenomed feelings, there was a nobleman of great distinction, for whose release very strenuous efforts had unavailingly been made. As a member of the National Institute, it was thought that Jenner, from his powerful influence, might, through that body, effect the object, and, accordingly, by request, he made the application. The Institute, however, was impotent without the emperor's approbation, and, therefore, addressed him on

---

* The above anecdote I give on the authority of a person entitled to confidence. Part of the statement I know to be true. In the fall of 1801, I was on Wimbleton Common, near London, on the occasion of the review, saw the duke exposed to a heavy rain for several hours, read the daily bulletins of his illness, and it was generally said about town that Mosely had been dismissed and degraded, for the very reasons mentioned. The anecdote is communicated to vindicate, not to insult, the memory of a man of no common merit.

the subject, whose memorable reply was: 'That, which I would not grant to the prayer of embodied Europe besides, I will concede to the wishes of the most illustrious benefactor of humanity. It shall be done.'" Y.

